# Associations between Size-Specific Particulate Matter and Lung Function: Results from the China Pulmonary Health Study

**DOI:** 10.1021/envhealth.5c00676

**Published:** 2026-06-02

**Authors:** Zijun Li, Xingyao Tang, Xueyan Han, Sinan Wu, Zhaoyang Pan, Linhong Pang, Hailu Zhu, Yicong Wang, Qi Wang, Xinran Shan, Ting Yang, Jing Wei, Tianjia Guan, Ke Huang, Chen Wang, The China Pulmonary Health (CPH) Study Group

**Affiliations:** † School of Population Medicine and Public Health, Chinese Academy of Medical Sciences & Peking Union Medical College, Beijing 100730, China; ‡ Department of Pulmonary and Critical Care Medicine, Center of Respiratory Medicine, China-Japan Friendship School of Clinical Medicine, Capital Medical University, Beijing 100029, China; § National Center for Respiratory Diseases, State Key Laboratory of Respiratory Health and Multimorbidity, National Clinical Research Center for Respiratory Diseases, Institute of Respiratory Medicine, Chinese Academy of Medical Sciences, Department of Pulmonary and Critical Care Medicine, Center of Respiratory Medicine, China-Japan Friendship Hospital, Beijing 100029, China; ∥ School of Health Policy and Management, Chinese Academy of Medical Sciences & Peking Union Medical College, Beijing 100730, China; ⊥ Institutes of Science and Development, Chinese Academy of Sciences, Beijing 100190, China; # National Center for Respiratory Medicine, State Key Laboratory of Respiratory Health and Multimorbidity, National Clinical Research Center for Respiratory Diseases, Institute of Respiratory Medicine, Chinese Academy of Medical Sciences, Department of Clinical Research and Data Management, Center of Respiratory Medicine, China-Japan Friendship Hospital, Beijing 100029, China; ∇ MEEKL-AERM, College of Environmental Sciences and Engineering, Institute of Tibetan Plateau, and College of Environmental Sciences and Engineering, Peking University, Beijing 100871, China

**Keywords:** particulate matter, particle size, lung function, PM_1_, coarse particle

## Abstract

Emerging evidence suggests that the respiratory health effects of particulate matter (PM) may vary by aerodynamic diameter. However, comprehensive investigations comparing the effects of size-specific PM on lung function indicators remain limited. We analyzed lung function data from the China Pulmonary Health (CPH) Study to assess the associations between long-term exposure to PM_1_, PM_1–2.5_, PM_2.5_, PM_2.5–10_, and PM_10_ and indicators of large and small airway function using multivariable linear models. This study included 50,991 adults aged 20 years and above. For large airway function, each interquartile range (IQR) increase in PM_1_, PM_1–2.5_, PM_2.5_, and PM_10_ was significantly associated with a decrease in FEV_1_, FVC, and FEV_1_/FVC. The associations between FEV_1_ and FVC strengthened as the particle size decreased. PM_1_ showed the strongest association, with the lowest FEV_1_ (−36.84 mL, 95% confidence interval (CI): −45.91, −27.76 mL) and FVC (−34.63 mL, 95% CI: −45.48, −23.79 mL) values. For small airway function, all size-specific particles were significantly associated with decreases in FEF_75_ and FEF_25–75_. Specifically, each IQR increase in PM_2.5–10_ exposure was associated with the largest reduction in the airway function indicators. Furthermore, stratified analyses showed stronger adverse associations in vulnerable subpopulations, including females, older adults, former smokers, biomass nonusers, and participants with chronic obstructive pulmonary disease (COPD). Size-specific PM exposure has differential health effects on both large and small airway lung function indicators. Therefore, it is essential to account for particle size in health risk assessments and the regulation of PM.

## Graphical abstract

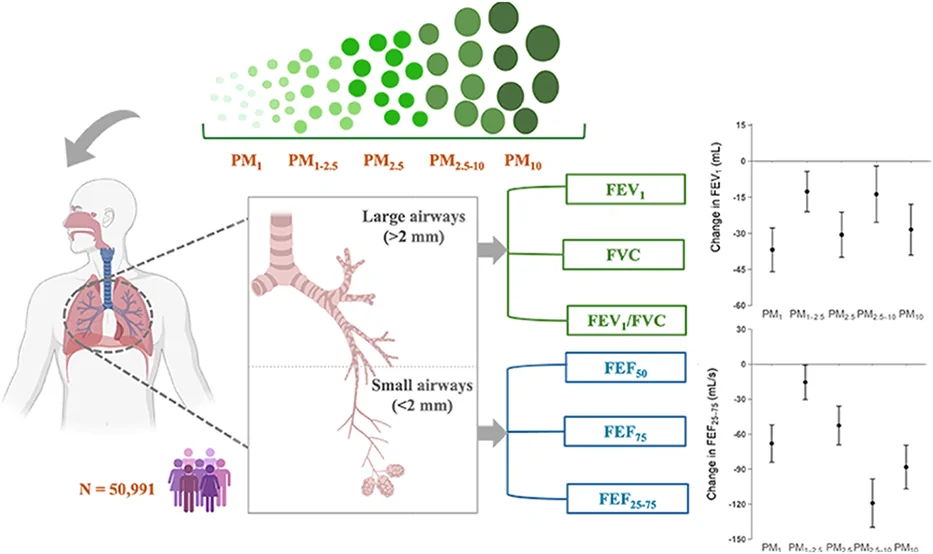

## 1 Introduction

Ambient particulate matter (PM) pollution has long posed a public health concern.^1^ Existing evidence has shown that PM with different aerodynamic diameters may have varying health effects.^2^ For respiratory health, particle size affects the regional deposition pattern and clearance mechanisms in the respiratory tract and thus influences the pathogenic potential of inhaled aerosols.^3^ Although numerous studies have investigated routinely monitored fractions like PM_2.5_ and PM_10_, the differential respiratory impacts of PM_1_, PM_1–2.5_, and PM_2.5–10_ have not been adequately studied.^4,5^

Lung function measurements serve as critical, objective indicators of pulmonary impairment and provide diagnostic and prognostic value for respiratory diseases and mortality.^6^ The associations between long-term exposure to ambient PM and lung function remain inconsistent across studies. While some European longitudinal studies have reported no significant associations,^7^ multiple large-scale studies have reported significant associations between PM exposure and decreases in lung function indicators across North America,^7^ Europe,^8^ and Asia.^9^ Moreover, because particle size is a key property of PM,^10^ the associations between differently sized particles and lung function remain unclear. Although toxicological evidence has demonstrated that smaller particles (e.g., PM_1_) induce stronger inflammatory and cytotoxic responses than larger particles do,^11^ epidemiological findings have shown limited and inconsistent support for this size-dependent toxicity in terms of lung function. A recent study in China (*n* = 1,592) revealed that long-term exposure to PM_1_ was more strongly associated with decreased lung function than exposure to PM_2.5_.^12^ A few large-scale investigations, such as the UK Biobank study (*n* = 303,887) and WHO SAGE (*n* = 11,693), have similarly reported that PM_2.5_ is more strongly linked to reduced pulmonary function than are PM_2.5–10_ or PM_10_.^8,13^ However, some smaller cohort studies (e.g., *n* = 1,241) have paradoxically reported stronger associations with larger particles (PM_10_ or PM_2.5–10_) than with PM_2.5_.^14^ Given these inconsistent findings, a large-population study is warranted to investigate the deleterious effects of size-specific PM on lung function indicators.

Most prior research has focused on the effects of PM on large airway function, whereas evidence on the associations between size-specific particles and small airway (less than 2 mm in diameter) indicators is lacking. Unlike large airways, small airways, which lack cartilaginous support and mucous glands, are more vulnerable to damage from inhaled toxic particles and gases.^15^ Abnormalities in lung function parameters related to small airways are considered sensitive indicators of asthma and chronic obstructive pulmonary disease (COPD).^16^ Owing to limited existing research, data on whether PM affects small airway function and how this effect varies with size-specific particles remain inconsistent and unclear.^17^ Therefore, investigating the effects of size-specific PM on small airways is necessary to determine how different-sized particles can affect the respiratory system at an early stage. Furthermore, few investigations of size-fractionated particles and adult lung function have been conducted in China, where PM pollution levels, size distributions, and chemical compositions differ markedly from those in developed countries.^18^ Moreover, since individual susceptibility to PM varies substantially,^4,8^ identifying vulnerable subpopulations under China’s unique pollution characteristics is essential for targeted interventions.

To address these gaps, we analyzed the associations between PM_1_, PM_1–2.5_, PM_2.5_, PM_2.5–10_, and PM_10_ and lung function using cross-sectional data obtained from the China Pulmonary Health (CPH) Study to evaluate how particle size influences respiratory health.

## 2 Methods

### 2.1 Study Participants and Design

The CPH study is a nationwide multicenter cross-sectional investigation that was conducted from June 2012 to May 2015. The study employed a multistage stratified cluster sampling approach to recruit a national sample of adults aged 20 years and older across ten provinces and autonomous regions in China. The detailed methodology regarding the survey design has been published previously.^19^ A total of 57,779 participants were initially enrolled; after excluding those who declined to participate and those with incomplete or ineligible spirometry results, 50,991 participants were included. This study was approved by the Institutional Review Boards of Capital Medical University (Approval No. 11-ke-42), and written informed consent was obtained from all participants.

### 2.2 Lung Function Assessment

Pulmonary function testing was administered by certified technicians utilizing MasterScreen Pneumo PC spirometers (CareFusion, Yorba Linda, CA, USA), with a 3-L syringe used for daily calibration.^20^ Spirometry was conducted with the participants seated, wearing nose clips, and using disposable mouthpieces. The participants performed up to eight forced expiratory maneuvers until two consecutive forced vital capacity (FVC) and forced expiratory volume in 1 s (FEV_1_) measurements differed by no more than 150 mL, ensuring reproducibility.

This study also evaluated forced expiratory flow at 50% of forced vital capacity (FEF_50_), forced expiratory flow at 75% of forced vital capacity (FEF_75_), and forced expiratory flow between 25% and 75% of forced vital capacity (FEF_25–75_). An expert panel reviewed all spirometric data for quality assurance on the basis of American Thoracic Society/European Respiratory Society (ATS/ERS) criteria and excluded tests that failed to meet established standards.^20^ We defined COPD status as a postbronchodilator FEV_1_/FVC < 0.70 according to the 2025 Global Initiative for Chronic Obstructive Lung Disease (GOLD) guidelines.^21^

### 2.3 Data Collection

Data were collected by trained investigators using a standardized questionnaire to obtain information on sociodemographic characteristics, smoking status, household fuel use patterns, personal respiratory disease history, and family history of respiratory conditions.

### 2.4 Exposure Assessment

The exposures of PM_1_, PM_2.5_, and PM_10_ were obtained from the ChinaHighAirPollutants (CHAP) data set (https://weijing-rs.github.io/product.html), which provides high-spatial-resolution (1 km × 1 km) data sets of ground-level air pollutants. The PM_1–2.5_ and PM_2.5–10_ concentrations were derived by subtracting PM_1_ from PM_2.5_ and subtracting PM_2.5_ from PM_10_, respectively. Exposures to surface ozone (O_3_) and nitrogen dioxide (NO_2_) were also obtained from the CHAP data set.^22–24^ Several epidemiological studies have employed the CHAP database to investigate associations between air pollution and health because of its high spatial–temporal resolution, public accessibility, and robust performance.^25^ Previous publications have detailed the methodology for calculating pollutant concentrations in the CHAP data sets.^26–28^ The CHAP data set derives its PM_1_, PM_2.5_, and PM_10_ concentrations from a mature, machine learning-based Space-Time Extremely Randomized Trees (STET) model, which integrates ground-based measurements, satellite aerosol optical depth (AOD), meteorological data, and ancillary information. This data set yielded high-quality results with a cross-validation coefficient of determination (CV-R^2^) of 0.83 for daily PM_1_,^26^ 0.92 for daily PM_2.5_,^27^ and 0.90 for daily PM_10_;^28^ it also yielded root-mean-square errors (RMSEs) of 9.5 μg/m^3^ for daily PM_1_, 10.76 μg/m^3^ for daily PM_2.5_, and 21.12 μg/m^3^ for daily PM_10_.^26–28^ Meteorological data, including temperature and relative humidity, were obtained from the fifth generation of European ReAnalysis (ERA5)-Land reanalysis data set with a spatial resolution of 0.1° × 0.1°. The CPH study collected exact residential addresses for all participants. Using geocoding techniques, we converted these addresses into geographical coordinates, which were then used to match the concentrations estimated for the 1 km × 1 km grid cell location. We estimated the annual mean PM_2.5_ and PM_10_ values for the 12 months preceding spirometry and the annual mean PM_1_ value for the 365 days before spirometry.

### 2.5 Statistical Analysis

Descriptive statistics for all participants included at baseline were summarized. We utilized multivariable linear regression models to examine the associations between lung function parameters and 1-year average PM exposure of varying sizes before the lung function test. The models were adjusted for age, sex, BMI (categorized as underweight (<18.5 kg/m^2^), normal (18.5–24.9 kg/m^2^), and overweight/obese (≥25 kg/m^2^)),^29^ education level (primary school or lower, middle or high school, college or above), geographic region (northeast, east, central, north, south, northwest, southwest), type of residence (urban/rural), smoking status (never smoker, current smoker, former smoker), biomass use (yes/no), number of household smokers (0, 1, ≥2), childhood history of pneumonia or bronchitis (yes/no), childhood history chronic cough (yes/no), season for conducting spirometry tests, and the calendar year of lung function testing. Nonlinear exposure–response relationships between size-specific particles and lung function were investigated using thin plate regression splines with three degrees of freedom.

According to methods described in previous studies, we calculated the PM_1_/PM_2.5_ and PM_1–2.5_/PM_2.5_ ratios using lag 1-year exposure levels to quantify the relative contributions of PM_1_ and PM_1–2.5_ to the risk of decreased lung function indicators associated with PM_2.5_; we also calculated the PM_2.5_/PM_10_ and PM_2.5–10_/PM_10_ ratios to assess their contributions to the corresponding risk associated with PM_10_.^30^

Subgroup analyses were conducted to examine potential effect modifications by age, sex, BMI, education level, biomass use, smoking history, residence type, and spirometry-defined COPD status. We employed two distinct sets of two-pollutant models to assess the robustness of the observed associations. First, to disentangle the effects of different particle size fractions, models were constructed as follows: models for PM_1_ were adjusted for PM_1–2.5_, models for PM_1–2.5_ were adjusted for PM_1_, models for PM_2.5_ were adjusted for PM_2.5–10_, and models for PM_2.5–10_ were adjusted for PM_2.5_. Second, to investigate potential confounding by gaseous copollutants, we separately adjusted the single-pollutant models for O_3_ and NO_2_. Furthermore, we conducted a sensitivity analysis where models were additionally adjusted for lag 1-year average temperature and relative humidity using natural spline functions.

All analyses were conducted using R statistical software (version 4.3.2; R Foundation for Statistical Computing). Two-tailed statistical tests were applied, with *p* value < 0.05 considered statistically significant.

## 3 Results

### 3.1 General Characteristics of the Study Participants and Environmental Variables

This study included 50,991 participants, and their demographic characteristics are presented in Table 1. The mean age of the participants was 49.38 years (standard deviation (SD) = 13.86), and 42.06% were males. A total of 35.77% of the participants were classified as overweight or obese (BMI ≥ 25 kg/m^2^). The majority of the participants lived in urban areas (64.48%). Additionally, 26.73% of the participants used biomass fuel, 22.99% were current smokers, and 5.57% were former smokers.

**Table 1:** Characteristics of the Study Participants from the China Pulmonary Health Study^a^

Characteristic	All participants
Age [years, mean (SD)]	49.38 (13.86)
Male [n (%)]	21446 (42.06)
BMI [n (%)]	
0–18.4 kg/m^2^	2055 (4.03)
18.5–24.9 kg/m^2^	30699 (60.20)
≥25 kg/m^2^	18237 (35.77)
Education level [n (%)]	
Primary school or lower	12755 (25.01)
Middle or high school	29170 (57.21)
College or above	9066 (17.78)
Geographic region [n (%)]	
Northeast	5131 (10.06)
East	10069 (19.75)
Central	5420 (10.63)
North	10332 (20.26)
South	4934 (9.68)
Northwest	5516 (10.82)
Southwest	9589 (18.81)
Residence [n (%)]	
Urban	32879 (64.48)
Rural	18112 (35.52)
Cigarette smoking [n (%)]	
Never-smoker	36429 (71.44)
Current smoker	11724 (22.99)
Former smoker	2838 (5.57)
Biomass fuel [n (%)]	13628 (26.73)
Smokers in the household [n (%)]	
0	1009 (1.98)
1	26759 (52.48)
≥2	23223 (45.54)
COPD [n (%)]	4908 (9.63)
History of pneumonia or bronchitis during childhood [n (%)]	2444 (4.79)
Chronic cough during childhood [n (%)]	1528 (3.00)
Parental history of respiratory diseases [n (%)]	5196 (10.19)
FEV_1_ [L, mean (SD)]	2.67 (0.74)
FVC [L, mean (SD)]	3.40 (0.89)
FEV_1_/FVC [%, mean (SD)]	78.53 (9.11)
FEF_50_ [L/s, mean (SD)]	3.18 (1.26)
FEF_75_ [L/s, mean (SD)]	1.06 (0.62)
FEF_25–75_ [L/s, mean (SD)]	2.29 (1.10)

a Abbreviations: BMI, body mass index; SD, standard deviation; n, number; COPD, chronic obstructive pulmonary disease; FEV_1_, forced expiratory volume in 1 s; FVC, forced vital capacity; FEF_50_, forced expiratory flow at 50% of forced vital capacity; FEF_75_, forced expiratory flow at 75% of forced vital capacity; FEF_25–75_, forced expiratory flow between 25% and 75% of forced vital capacity.

The 1-year mean concentration of PM_1_ before the lung function test was 35.81 μg/m^3^. The mean concentration of PM_1–2.5_ was 26.76 μg/m^3^. Additionally, the mean concentrations of PM_2.5_, PM_2.5–10_, and PM_10_ were 62.58, 41.91, and 104.48 μg/m^3^, respectively. Furthermore, the mean concentrations of O_3_ and NO_2_ were 82.88 μg/m^3^ and 38.32 μg/m^3^, as presented in Table 2.

**Table 2:** Summary Statistics of Lag 1-Year Environmental Exposures^a^

Pollutants	Mean	SD	Min	P_25_	P_50_	P_75_	Max	IQR
PM_1_	35.81	8.99	17.98	28.38	35.96	41.66	57.64	13.28
PM_1–2.5_	26.76	6.29	12.44	23.15	27.56	31.54	40.81	8.39
PM_2.5_	62.58	14.65	30.42	51.42	63.64	72.99	96.27	21.57
PM_2.5–10_	41.91	14.15	17.41	31.62	41.02	50.03	73.79	18.42
PM_10_	104.48	26.92	48.96	86.28	104.08	122.83	162.54	36.55
O_3_	82.88	8.76	60.83	76.90	82.67	89.18	100.69	12.28
NO_2_	38.32	11.04	15.88	30.65	36.92	45.38	63.74	14.73
Temperature	14.34	4.14	5.58	10.93	15.38	17.34	23.90	6.41
Humidity	68.03	10.79	46.1	59.27	71.64	76.44	83.19	17.18

a Abbreviations: P_25_, the 25th percentile; P_50_, the 50th percentile; P_75_, the 75th percentile; SD, standard deviation; IQR, interquartile range; concentrations for PM (particulate matter), O_3_ (ozone), and NO_2_ (nitrogen dioxide) are in μg/m^3^; temperature in degrees Celsius (°C); Relative humidity in percent (%). PM_1_, particulate matter with an aerodynamic diameter less than or equal to 1 μm; PM_1–2.5_, particulate matter with an aerodynamic diameter between 1 and 2.5 μm; PM_2.5_, particulate matter with an aerodynamic diameter less than or equal to 2.5 μm; PM_2.5–10_, particulate matter with an aerodynamic diameter between 2.5 and 10 μm; PM_10_, particulate matter with an aerodynamic diameter less than or equal to 10 μm.

### 3.2 Associations between Size-Specific PM and Lung Function

Figure 1 illustrates the associations between lung function parameters and the concentrations of PM_1_, PM_1–2.5_, PM_2.5_, PM_2.5–10_, and PM_10_, with the interquartile range (IQR) increments shown. We consistently observed significant associations between increased PM_1_, PM_1–2.5_, PM_2.5_, PM_2.5–10_, and PM_10_ levels and decreases in FEV_1_, FEV_1_/FVC, FEF_75_, and FEF_25–75_ values. PM_1_, PM_1–2.5_, PM_2.5_, and PM_10_ were significantly associated with FVC, whereas PM_2.5–10_ was not significantly associated with FVC. Additionally, PM_1_, PM_2.5_, PM_2.5–10_, and PM_10_ were significantly associated with FEF_50_, but PM_1–2.5_ was not significantly associated with FEF_50_.

**Figure 1:**
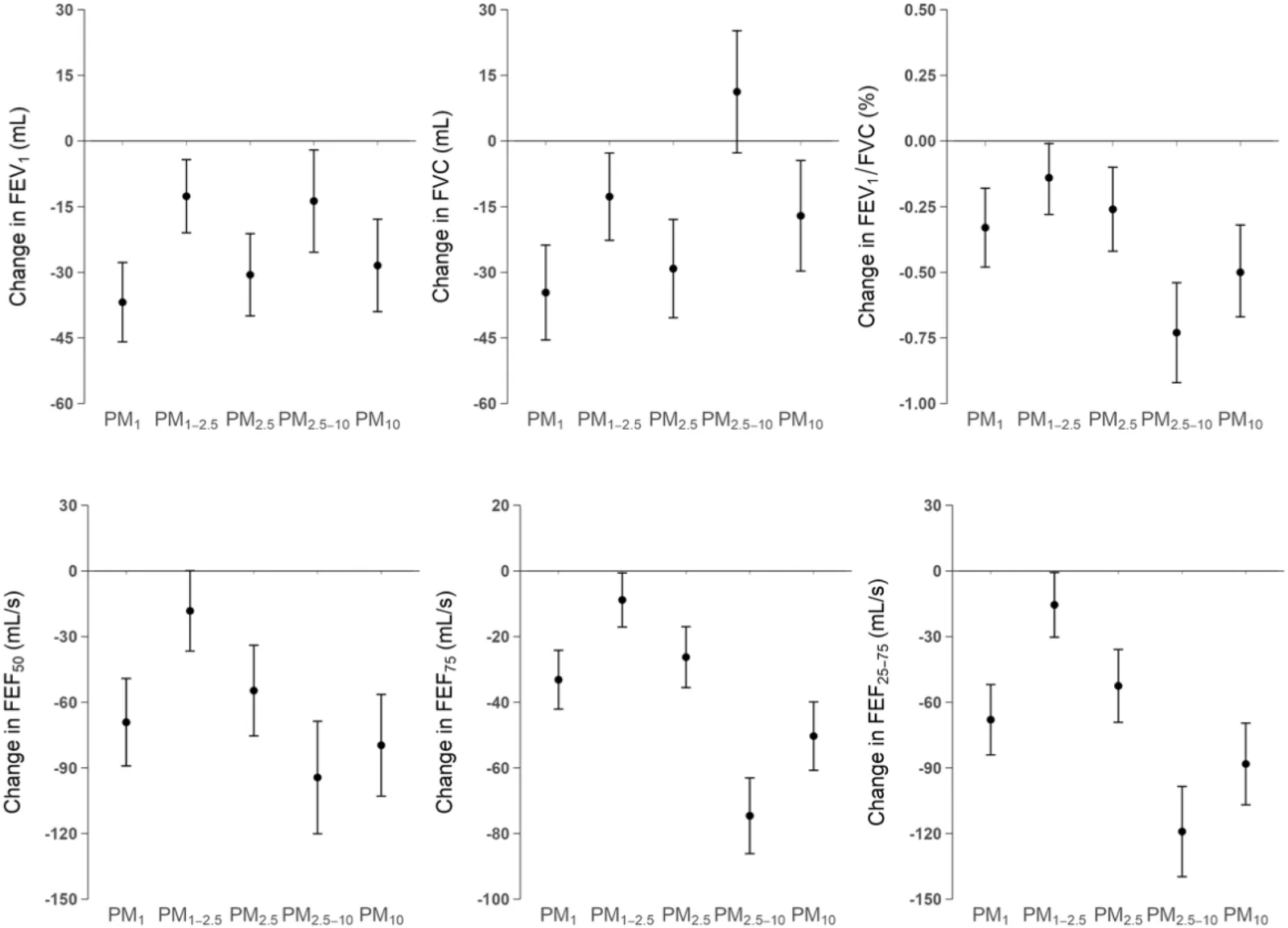
Mean difference (95% CI) in lung function measures associated with each IQR (μg/m^3^) increase in lag 1-year size-specific particulate matter. The model was adjusted for age, sex, education, marriage, occupation, BMI, smoking status, number of smokers in the household, biomass use, season for lung function test, geographic region, residence, history of pneumonia or bronchitis during childhood, chronic cough during childhood, and parental history of respiratory diseases. Abbreviations are the same as Tables 1 and 2.

In terms of large airway lung function parameters, each IQR increase in the PM_1_ concentration was associated with lower FEV_1_ (−36.84 mL, 95% CI (confidence interval): −45.91, −27.76 mL) and FVC (−34.63 mL, 95% CI: −45.48, −23.79 mL) values. The associations between PM and FEV_1_, as well as FVC, strengthened as the particle size decreased. However, the most significant decrease in FEV_1_/FVC was observed for PM_2.5–10_ (−0.73%, 95% CI: −0.92, −0.54%), followed by PM_1_ (−0.33%, 95% CI: −0.48, −0.18%). Among the small airway lung function parameters, PM_2.5–10_ had the most pronounced health effects. Each IQR increase in the PM_2.5–10_ concentration was associated with lower FEF_50_ (−94.38 mL/s, 95% CI: −120.09, −68.68 mL/s), FEF_75_ (−74.57 mL/s, 95% CI: −86.09, −63.05 mL/s), and FEF_25–75_ (−119.09 mL/s, 95% CI: −139.71, −98.47 mL/s) values.

A significant association was found between the PM_1_/PM_2.5_ ratio and lung function. Specifically, per 1% increase in the PM_1_/PM_2.5_ ratio, decreases were observed in FEV_1_ (−2.59 mL, 95% CI: −3.90, −1.27 mL), FVC (−3.41 mL, 95% CI: −4.98, −1.83 mL), FEV_1_/FVC (−0.05%, 95% CI: −0.07, −0.02%), FEF_50_ (−9.52 mL/s, 95% CI: −12.79, −6.25 mL/s), FEF_75_ (−6.71 mL/s, 95% CI: −8.18, −5.24 mL/s), and FEF_25–75_ (−10.83 mL/s, 95% CI: −13.46, −8.21 mL/s) values. In contrast, a 1% increase in the PM_2.5_/PM_10_ ratio was associated with decreases in only the large airway lung function indicators FEV_1_ and FVC. The PM_2.5–10_/PM_10_ ratio was associated with decreases in the small airway lung function parameters FEF_50_, FEF_75_, and FEF_25–75_, with values of −4.32 mL/s (95% CI: −7.45, −1.19 mL/s), −6.54 mL/s (95% CI: −7.94, −5.13 mL/s), and −8.70 mL/s (95% CI: −11.21, −6.18 mL/s), respectively (Table S1).

### 3.3 Exposure–Response Relationships

Figure 2 presents the exposure–response relationships for size-specific particles with FEV_1_, whereas Figures S1–S5 show those relationships for FVC, FEV_1_/FVC, FEF_50_, FEF_75_, and FEF_25–75_. For large airway parameters (FEV_1_, FVC, and FEV_1_/FVC), the spline curves revealed nonlinear patterns. As exemplified by FEV_1_, following slight initial fluctuations at low concentrations, sharp declines were consistently observed when exposures exceeded approximately 35 μg/m^3^ for PM_1_, 60 μg/m^3^ for PM_2.5_, and 100 μg/m^3^ for PM_10_. Additionally, the PM_1–2.5_ fraction exhibited a continuous linear decline without a threshold, whereas PM_2.5–10_ demonstrated an accelerating downward trend at higher concentrations. For all small airway indicators (FEF_50_, FEF_75_, and FEF_25–75_), consistent downward relationships were observed across the exposure range.

**Figure 2:**
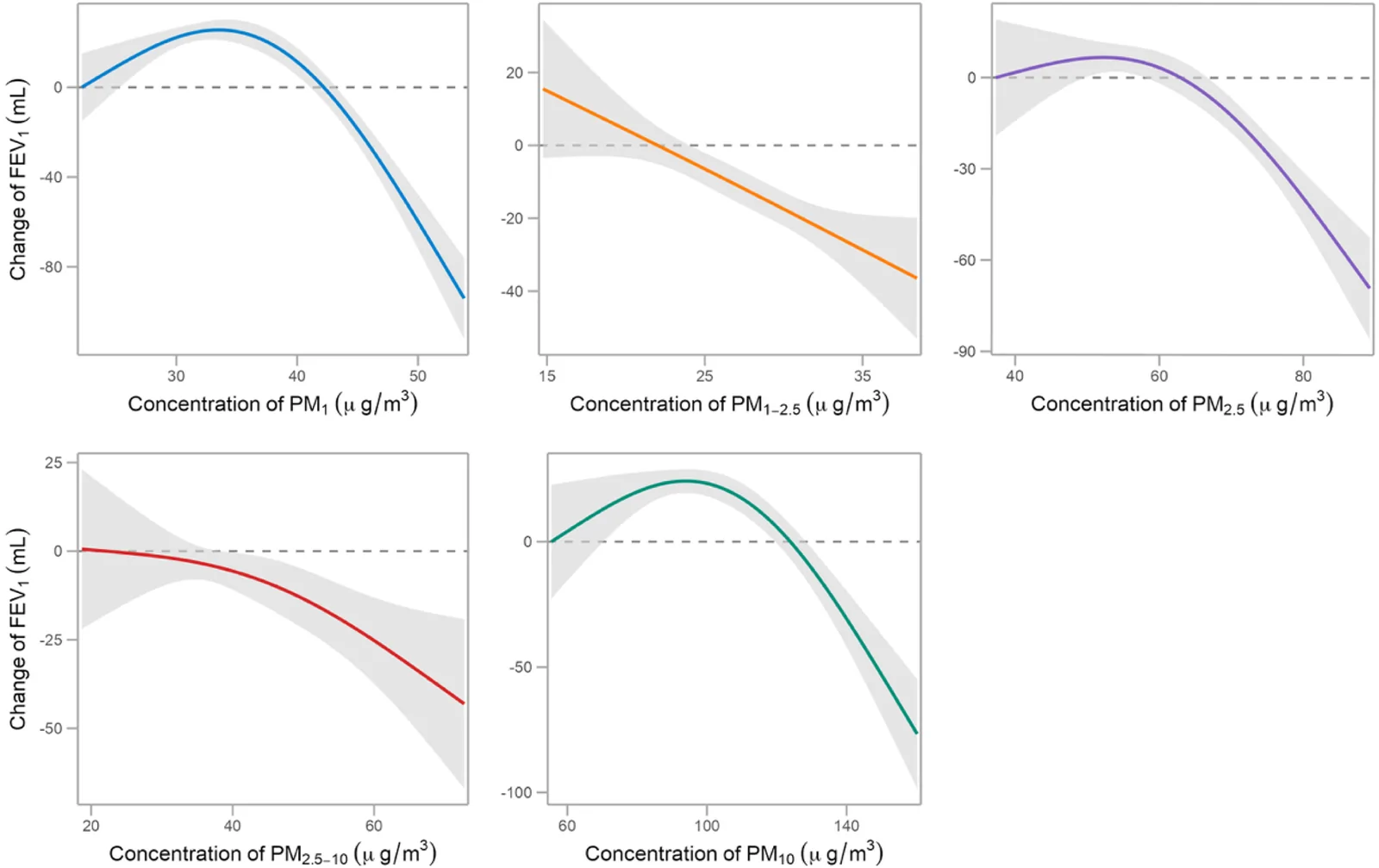
Exposure–response curves for the relationships of lag 1-year size-specific particulate matter concentrations with changes in FEV_1_ (mL). Abbreviations: FEV_1_, forced expiratory volume in 1 s.

### 3.4 Stratified Analysis

The results of the stratification analysis are presented in Figures S6 and S7. Sex, biomass use, and smoking history were identified as effect modifiers in the associations between size-specific PM exposure and FEV_1_. Specifically, females, biomass nonusers, and former smokers were found to be more susceptible to the adverse effects of PM exposure (*p* for interaction < 0.05). Additionally, age and biomass use significantly interacted with size-specific particles in relation to FEF_25–75_, with older individuals and biomass nonusers showing greater susceptibility. Inconsistent stratified results were observed for BMI, education level, residence type, and COPD status across different particle sizes. In general, participants with higher BMIs, lower education levels, those living in urban areas, and those with COPD were more susceptible to variable PM exposure.

### 3.5 Sensitivity and Two-Pollutant Model Analyses

In two-pollutant models adjusted for gaseous pollutants (Table S2), the adverse associations of PM with lung function were generally robust to adjustment for O_3_ but were attenuated when controlling for NO_2_. Notably, even after these adjustments, PM_1_ consistently showed the strongest significant associations with the large airway indicators FEV_1_ and FVC, while PM_2.5–10_ exhibited the largest effects on small airway indicators (FEF_50_, FEF_75_, and FEF_25–75_). When mutually adjusting for PM fractions (Figure S8), the associations for PM_1_ were stable after controlling for PM_1–2.5_. The effects of PM_2.5_ on FEV_1_ and FVC also remained stable when adjusted for PM_2.5–10_. In contrast, the associations of PM_2.5–10_ with FEV_1_/FVC and small airway indicators were robust to adjustment for PM_2.5_, while its associations with FEV_1_ and FVC became nonsignificant. Additionally, the main findings remained largely robust in sensitivity analyses that adjusted for temperature and humidity (Table S3).

## 4 Discussion

Our findings demonstrate that size-specific PM exposure is associated with impaired lung function, with distinct patterns observed across different airway regions. For large airways, we observed that health effects increased as particle size decreased, with PM_1_ demonstrating the strongest adverse effects. The PM_1_/PM_2.5_ ratio confirmed the significant contribution of PM_1_ to PM_2.5_-associated large airway dysfunction. In contrast, all the PM fractions, particularly PM_2.5–10_, which had the largest effect size, had significantly small airway effects. The PM_2.5–10_/PM_10_ ratio further demonstrated the dominant role of coarse particles in PM_10_-related small airway dysfunction.

Our study is the first epidemiological study to comprehensively evaluate the detailed classifications of particle sizes regarding the lung functions of both large and small airways in adults. For large airways, our results align with previous research in Chinese adults, confirming that PM_1_ exerts the strongest adverse effect on FVC compared to PM_2.5_ and PM_10_ (e.g., a 122.1 mL vs 54.6 and 42.9 mL decrease per 5 μg/m^3^ increase, respectively).^12^ However, our findings contrast with those from the European Study of Cohorts for Air Pollution Effects (ESCAPE),^5^ which identified PM_10_ as the fraction with the greatest influence on lung function. This variation could originate from geographical differences in PM constituents, long-term air pollution levels, population susceptibility, and different lengths of study periods.^31^ Our study, which is consistent with that of Liu et al.,^12^ revealed that the FEV_1_/FVC ratio increases with higher air pollution levels. Since the development of airflow obstruction is characterized by a slowly progressive decline in FEV_1_ relative to FVC,^6^ this increase does not signify an improvement in pulmonary function. In contrast to the findings related to large airways, small airway function was significantly impaired across all PM size fractions, with PM_2.5–10_ demonstrating the largest effect, followed by PM_1_. This observation aligns with a cohort study that reported that coarse particulate matter had the most substantial impact on FEF_25–75_ in elderly individuals.^14^ Going beyond previous studies that mostly relied on linear assumptions, our exposure-response analysis revealed distinct curve shapes across different airway regions. For large-airway indicators, we observed a nonlinear association characterized by a “levelling-off” effect at higher exposure ranges, a phenomenon noted in other air pollution studies.^9^ Consequently, linear association models may be overly simplistic for these parameters. Furthermore, we observed distinct exposure-response patterns characterized by nonlinear threshold effects in large airways and continuous linear declines in small airways. These distinct curve shapes likely reflect that small airways lack such compensatory reserves and exhibit immediate vulnerability to particulate toxicity.^15,32^

The distinct pathological responses of large and small airways to size-specific PM are driven by differential deposition efficiency and size-dependent chemical toxicity. Physically, fine particles penetrate deeply into the peripheral lung, while coarse particles (PM_2.5–10_) deposit proximally via inertial impaction or reach distal small airways through sedimentation.^33^ Mechanistically, particle size dictates chemical composition and subsequent damage pathways.^34–36^ Fine particles are highly enriched in redox-active organic compounds (e.g., polycyclic aromatic hydrocarbons) and transition metals (e.g., Cu, Fe, V).^37^ These specific constituents exert profound chemical toxicity by elevating oxidative potential (OP) and driving reactive oxygen species (ROS) overproduction, which induces widespread airway epithelial damage.^38^ Conversely, the impact of PM_2.5–10_ is primarily driven by its unique enrichment in crustal elements (e.g., Si, Al) and biogenic materials (e.g., endotoxins).^39^ These coarse constituents directly activate alveolar macrophages to release pro-inflammatory cytokines, triggering intense localized inflammation, mucus hypersecretion, and structural airway narrowing.^40^

Subgroup analyses revealed that females, the elderly, former smokers, biomass nonusers, living in urban areas, and patients with COPD were particularly vulnerable to particulate matter-related lung function decline. The heightened susceptibility in females and the elderly may be attributed to sex-specific inflammatory pathways and age-related physiological vulnerabilities, respectively.^41^ The elevated risk in former smokers could stem from a combination of lasting epithelial damage and a restored inflammatory response, unlike the blunted sensitivity potentially seen in current smokers.^42^ As expected, patients with COPD face greater risks due to preexisting conditions like impaired mucociliary clearance.^43^ Intriguingly, biomass nonusers also showed increased susceptibility, possibly indicating a lack of the adaptive tolerance seen in those chronically exposed to indoor pollutants—a hypothesis that warrants further investigation. We observed that urban residents showed greater susceptibility to the adverse effects of PM on FEV_1_. This heightened susceptibility potentially stems from differential toxicity due to varied PM composition,^44^ disparities in population susceptibility driven by socioeconomic status,^4^ and the inequity in the distribution of regulatory PM monitors.^45^

Our study provides several new insights. While the WHO global air quality guidelines primarily target PM_2.5_ and PM_10_ mass concentrations,^46^ our findings demonstrate that this approach may be insufficient as it overlooks the differential toxicity of various size fractions. This highlights the need for a paradigm shift from purely mass-based regulations to more nuanced, health-effects-based strategies. Identifying the most hazardous PM size fractions allows for the development of more targeted and cost-effective mitigation policies. This study has several limitations. First, our cross-sectional data (2012–2015) precede China’s recent clean air policies, meaning absolute PM concentrations, chemical profiles, and their associated toxicities have since evolved.^47,48^ While extrapolating exact effect magnitudes to current profiles requires caution, evidence indicates that despite declining pollution levels, the effect size per unit increase in PM concentrations remains stable for respiratory outcomes.^49^ This suggests our findings remain critical for current risk assessments. Second, the health effects of ultrafine particulate matter were not evaluated. Third, relying on residential outdoor PM concentrations introduces nondifferential exposure misclassification by ignoring indoor exposures and individual mobility. However, this typically biases estimates toward the null, suggesting our findings may actually underestimate the true associations.^50^ Fourth, small sample sizes in certain strata (e.g., former smokers and low BMI participants) reduced statistical power and widened confidence intervals, warranting cautious interpretation of these subgroup results.

## 5 Conclusions

PM has distinct health effects on large and small airways, with smaller particles (PM_1_) being more detrimental to large airways and coarse particles (PM_2.5–10_) having the most pronounced impact on small airways. These findings challenge the adequacy of one-size-fits-all regulatory standards and underscore the importance of developing size-specific management strategies. An effective public health approach should consider not only the total mass of PM but also the relative contribution and toxicity of its different size fractions. By doing so, policymakers can better protect respiratory health and optimize the public health benefits of air pollution control measures. Further research is needed to better understand the differential health effects of size-specific PM and to translate these scientific insights into formal regulatory guidelines.

## Supplementary Material



## Notes

Tables and figures detailing the associations between PM ratios and lung function parameters; exposure-response relationships for lung function indicators; stratified analyses; results from two-pollutant and sensitivity models (PDF)
