# Supplementary material for: Associations between Size-Specific Particulate Matter and Lung Function: Results from the China Pulmonary Health Study
Source: Environ Health (Wash). 2026 Jun 2;4(10):2232–41. doi: 10.1021/envhealth.5c00676 (PMC13681440; doi:10.1021/envhealth.5c00676)
Supplement: Supplementary file 1 [file eh5c00676_si_001.pdf]

Supporting Information

Associations between Size-Specific Particulate Matter and Lung Function: Results from the China Pulmonary Health Study

Zijun Li,[1†] Xingyao Tang,[2,3†] Xueyan Han,[4,5] Sinan Wu,[6] Zhaoyang Pan,[4] Linhong Pang,[4] Hailu Zhu,[4] Yicong Wang,[4] Qi Wang,[4] Xinran Shan,[4] Ting Yang,[3#] Jing Wei,[7] Tianjia Guan,[4*] Ke Huang,[3*] Chen Wang[1,3*], and the China Pulmonary Health (CPH) Study Investigators

1 School of Population Medicine and Public Health, Chinese Academy of Medical Sciences & Peking Union Medical College, Beijing 100730, China.

2 Department of Pulmonary and Critical Care Medicine, Center of Respiratory Medicine, China-Japan Friendship School of Clinical Medicine, Capital Medical University, Beijing 100029, China.

3 National Center for Respiratory Diseases; State Key Laboratory of Respiratory Health and Multimorbidity; National Clinical Research Center for Respiratory Diseases; Institute of Respiratory Medicine, Chinese Academy of Medical Sciences; Department of Pulmonary and Critical Care Medicine, Center of Respiratory Medicine, China-Japan Friendship Hospital, Beijing 100029, China.

4 School of Health Policy and Management, Chinese Academy of Medical Sciences & Peking Union Medical College, Beijing 100730, China.

5 Institutes of Science and Development, Chinese Academy of Sciences, Beijing 100190, China.

6 National Center for Respiratory Medicine, State Key Laboratory of Respiratory Health and Multimorbidity, National Clinical Research Center for Respiratory Diseases, Institute of Respiratory Medicine, Chinese Academy of Medical Sciences, Department of Clinical Research and Data Management, Center of Respiratory Medicine, China-Japan Friendship Hospital, Beijing 100029, China

7 MEEKL-AERM, College of Environmental Sciences and Engineering, Institute of Tibetan Plateau, and College of Environmental Sciences and Engineering, Peking University, Beijing 100871, China.

† Zijun Li and Xingyao Tang contributed equally to this work.

# Senior author.

* Corresponding authors: Tianjia Guan, School of Health Policy and Management, Chinese Academy of Medical Sciences & Peking Union Medical College, Beijing 100730, China (gtj@sph.pumc.edu.cn); Ke Huang, National Center for Respiratory Diseases, State Key Laboratory of Respiratory Health and Multimorbidity, National Clinical Research Center for Respiratory Diseases, Institute of Respiratory Medicine, Chinese Academy of Medical Sciences, Department of Pulmonary and Critical Care Medicine, Center of Respiratory Medicine, China-Japan Friendship Hospital, Beijing 100029, China (huangke_zryy@163.com); Chen Wang, School of Population Medicine and Public Health, Chinese Academy of Medical Sciences & Peking Union Medical College, Beijing 100730, China, and National Center for Respiratory Diseases, State Key Laboratory of Respiratory Health and Multimorbidity, National Clinical Research Center for Respiratory Diseases, Institute of Respiratory Medicine, Chinese Academy of Medical Sciences, Department of Pulmonary and Critical Care Medicine, Center of Respiratory Medicine, China-Japan Friendship Hospital, Beijing 100029, China (wangchen@pumc.edu.cn).

Table S1. Mean difference (95% CIs) in lung function measures associated with the ratios of PM_1 to $PM_{2.5}$, $PM_{1-2.5}$ to $PM_{2.5}$, $PM_{2.5}$ to PM_{10}, and $PM_{2.5-10}$ to PM_{10}.

	$PM_1/PM_{2.5}$ [a]	$PM_{1-2.5}/PM_{2.5}$ [a]	$PM_{2.5}/PM_{10}$ [b]	$PM_{2.5-10}/PM_{10}$ [b]
FEV_1 (mL)	-2.59 (-3.90, -1.27)	2.99 (1.64, 4.34)	-5.32 (-6.62, -4.03)	3.65 (2.23, 5.07)
FVC (mL)	-3.41 (-4.98, -1.83)	2.12 (0.51, 3.73)	-8.09 (-9.64, -6.54)	7.69 (6.00, 9.39)
FEV_1/FVC (%)	-0.05 (-0.07, -0.02)	0.05 (0.02, 0.07)	0.07 (0.05, 0.10)	-0.07 (-0.10, -0.05)
FEF_{50} (mL/s)	-9.52 (-12.79, -6.25)	8.07 (5.10, 11.03)	4.32 (1.19, 7.45)	-4.32 (-7.45, -1.19)
FEF_{75} (mL/s)	-6.71 (-8.18, -5.24)	4.31 (2.98, 5.64)	6.54 (5.13, 7.94)	-6.54 (-7.94, -5.13)
FEF_{25-75} (mL/s)	-10.83 (-13.46, -8.21)	9.10 (6.72, 11.49)	8.70 (6.18, 11.21)	-8.70 (-11.21, -6.18)

β (95% CI) were estimated for per 1% increase in the ratio of PM_1 to $PM_{2.5}$, $PM_{1-2.5}$ to $PM_{2.5}$, $PM_{2.5}$ to PM_{10}, and the ratio of $PM_{2.5-10}$ to PM_{10}. The model was adjusted for age, sex, education, marriage, occupation, BMI, smoking history, smoking exposure, number of smokers in the household, biomass use, season for lung function test, geographic region, residence, history of pneumonia or bronchitis during childhood, chronic cough during childhood, and parental history of respiratory diseases.

[a] The model was adjusted for the concentration of $PM_{2.5}$.

[b] The model was adjusted for the concentration of PM_{10}.

Abbreviations: FEV_1, forced expiratory volume in 1 second; FVC, forced vital capacity; FEF_{50}, forced expiratory flow at the 50th percentile of forced vital capacity; FEF_{75}, forced expiratory flow at the 75th percentile of forced vital capacity; FEF_{25-75}, forced expiratory flow between 25% and 75% of forced vital capacity; PM_1, particulate matter with an aerodynamic diameter less than or equal to 1 μm; $PM_{1-2.5}$, particulate matter with an aerodynamic diameter between 1 and 2.5 μm; $PM_{2.5}$, particulate matter with an aerodynamic diameter less than or equal to 2.5 μm; $PM_{2.5-10}$, particulate matter with an aerodynamic diameter between 2.5 and 10 μm; PM_{10}, particulate matter with an aerodynamic diameter less than or equal to 10 μm.

Table S2. Associations of size-specific PM with lung function in single-pollutant models and models adjusted for gaseous co-pollutants.

Indicator	Primary Pollutant	Single-PollutantModel	Two-Pollutant Models	
			Adjusted for O_3	Adjusted for NO_2
		β (95% CI)	β (95% CI)	β (95% CI)
FEV_1	PM_1	-36.84 (-45.91, -27.76)	-42.38 (-51.80, -32.96)	-28.9 (-40.05, -17.74)
	$PM_{1-2.5}$	-12.63 (-20.97, -4.28)	-17.17 (-26.05, -8.29)	-0.22 (-9.63, 9.2)
	$PM_{2.5}$	-30.57 (-39.97, -21.17)	-37.34 (-47.27, -27.41)	-18.99 (-30.48, -7.5)
	$PM_{2.5-10}$	-13.73 (-25.42, -2.05)	-14.40 (-26.10, -2.69)	7.2 (-5.84, 20.24)
	PM_{10}	-28.44 (-39.00, -17.87)	-32.93 (-43.85, -22.02)	-10.84 (-23.91, 2.22)
FVC	PM_1	-34.63 (-45.48, -23.79)	-47.14 (-58.36, -35.92)	-25.73 (-38.99, -12.48)
	$PM_{1-2.5}$	-12.72 (-22.68, -2.76)	-25.79 (-36.36, -15.22)	0.76 (-10.43, 11.94)
	$PM_{2.5}$	-29.16 (-40.39, -17.93)	-44.79 (-56.61, -32.96)	-16.36 (-30.02, -2.71)
	$PM_{2.5-10}$	11.23 (-2.72, 25.18)	8.22 (-5.72, 22.15)	8.51 (-5.15, 22.17)
	PM_{10}	-17.09 (-29.71, -4.47)	-28.32 (-41.32, -15.32)	-7.85 (-22.85, 7.15)
FEV_1/FVC	PM_1	-0.33 (-0.48, -0.18)	-0.20 (-0.36, -0.04)	-0.29 (-0.48, -0.1)
	$PM_{1-2.5}$	-0.14 (-0.28, -0.01)	0.09 (-0.06, 0.24)	-0.01 (-0.17, 0.15)
	$PM_{2.5}$	-0.26 (-0.42, -0.10)	-0.09 (-0.26, 0.07)	-0.19 (-0.39, 0)
	$PM_{2.5-10}$	-0.73 (-0.92, -0.54)	-0.68 (-0.87, -0.48)	-0.71 (-0.93, -0.49)
	PM_{10}	-0.50 (-0.67, -0.32)	-0.36 (-0.55, -0.18)	-0.51 (-0.73, -0.29)
FEF_{50}	PM_1	-69.15 (-89.13, -49.16)	-69.43 (-90.23, -48.64)	-45.28 (-69.96, -20.6)
	$PM_{1-2.5}$	-18.25 (-36.62, 0.12)	-14.07 (-33.67, 5.52)	11.05 (-9.77, 31.87)
	$PM_{2.5}$	-54.68 (-75.39, -33.98)	-54.32 (-76.24, -32.41)	-23.15 (-48.57, 2.26)
	$PM_{2.5-10}$	-94.38 (-120.09, -68.68)	-92.83 (-118.64, -67.02)	-65.01 (-93.84, -36.18)
	PM_{10}	-79.66 (-102.92, -56.41)	-79.45 (-103.53, -55.36)	-50.56 (-79.45, -21.67)
FEF_{75}	PM_1	-33.15 (-42.11, -24.19)	-31.69 (-41.01, -22.37)	-20.72 (-31.76, -9.68)
	$PM_{1-2.5}$	-8.83 (-17.07, -0.59)	-5.07 (-13.86, 3.71)	5.3 (-4.01, 14.61)
	$PM_{2.5}$	-26.26 (-35.54, -16.97)	-24.14 (-33.96, -14.31)	-10.45 (-21.82, 0.91)
	$PM_{2.5-10}$	-74.57 (-86.09, -63.05)	-73.79 (-85.35, -62.24)	-66.91 (-79.8, -54.03)
	PM_{10}	-50.31 (-60.74, -39.88)	-49.58 (-60.37, -38.79)	-41.83 (-54.75, -28.91)
FEF_{25-75}	PM_1	-67.98 (-84.04, -51.92)	-79.02 (-95.72, -62.31)	-59.11 (-78.93, -39.29)
	$PM_{1-2.5}$	-15.47 (-30.23, -0.72)	-24.17 (-39.90, -8.43)	6.64 (-10.07, 23.35)
	$PM_{2.5}$	-52.53 (-69.16, -35.90)	-65.77 (-83.37, -48.17)	-34.71 (-55.11, -14.3)
	$PM_{2.5-10}$	-119.09 (-139.71, -98.47)	-122.37 (-143.08, -101.66)	-113.12 (-136.23, -90.01)
	PM_{10}	-88.20 (-106.87, -69.54)	-100.51 (-119.83, -81.18)	-83.69 (-106.86, -60.51)

Table S3. Sensitivity analysis of the associations between size-specific PM and lung function, adjusting for temperature and humidity

Indicator	Pollutant	Main Model<br><br>β (95% CI)	Model Adjusted for Temperature and Humidity)<br>β (95% CI)
FEV_1	PM_1	-36.84 (-45.91, -27.76)	-37.68 (-52.25, -23.10)
	$PM_{1-2.5}$	-12.63 (-20.97, -4.28)	-10.24 (-20.91, 0.42)
	$PM_{2.5}$	-30.57 (-39.97, -21.17)	-30.61 (-45.17, -16.05)
	$PM_{2.5-10}$	-13.73 (-25.42, -2.05)	-29.96 (-47.79, -12.13)
	PM_{10}	-28.44 (-39.00, -17.87)	-37.14 (-53.87, -20.41)
FVC	PM_1	-34.63 (-45.48, -23.79)	-44.44 (-61.78, -27.11)
	$PM_{1-2.5}$	-12.72 (-22.68, -2.76)	-19.33 (-32.02, -6.64)
	$PM_{2.5}$	-29.16 (-40.39, -17.93)	-41.37 (-58.70, -24.04)
	$PM_{2.5-10}$	11.23 (-2.72, 25.18)	-22.97 (-44.19, -1.75)
	PM_{10}	-17.09 (-29.71, -4.47)	-42.42 (-62.33, -22.51)
FEV_1/FVC	PM_1	-0.33 (-0.48, -0.18)	-0.10 (-0.34, 0.15)
	$PM_{1-2.5}$	-0.14 (-0.28, -0.01)	0.19 (0.01, 0.37)
	$PM_{2.5}$	-0.26 (-0.42, -0.10)	0.08 (-0.17, 0.32)
	$PM_{2.5-10}$	-0.73 (-0.92, -0.54)	-0.37 (-0.66, -0.07)
	PM_{10}	-0.50 (-0.67, -0.32)	-0.10 (-0.38, 0.18)
FEF_{50}	PM_1	-69.15 (-89.13, -49.16)	-54.52 (-86.72, -22.31)
	$PM_{1-2.5}$	-18.25 (-36.62, 0.12)	11.72 (-11.85, 35.29)
	$PM_{2.5}$	-54.68 (-75.39, -33.98)	-25.02 (-57.21, 7.17)
	$PM_{2.5-10}$	-94.38 (-120.09, -68.68)	-66.58 (-106.00, -27.17)
	PM_{10}	-79.66 (-102.92, -56.41)	-49.04 (-86.02, -12.05)
FEF_{75}	PM_1	-33.15 (-42.11, -24.19)	-30.57 (-45.00, -16.15)
	$PM_{1-2.5}$	-8.83 (-17.07, -0.59)	3.69 (-6.86, 14.24)
	$PM_{2.5}$	-26.26 (-35.54, -16.97)	-16.13 (-30.54, -1.71)
	$PM_{2.5-10}$	-74.57 (-86.09, -63.05)	-68.23 (-85.87, -50.59)
	PM_{10}	-50.31 (-60.74, -39.88)	-42.84 (-59.40, -26.28)
FEF_{25-75}	PM_1	-67.98 (-84.04, -51.92)	-53.19 (-79.01, -27.37)
	$PM_{1-2.5}$	-15.47 (-30.23, -0.72)	7.22 (-11.69, 26.12)
	$PM_{2.5}$	-52.53 (-69.16, -35.90)	-27.48 (-53.29, -1.67)
	$PM_{2.5-10}$	-119.09 (-139.71, -98.47)	-110.59 (-142.18, -79.01)
	PM_{10}	-88.20 (-106.87, -69.54)	-70.46 (-100.10, -40.82)

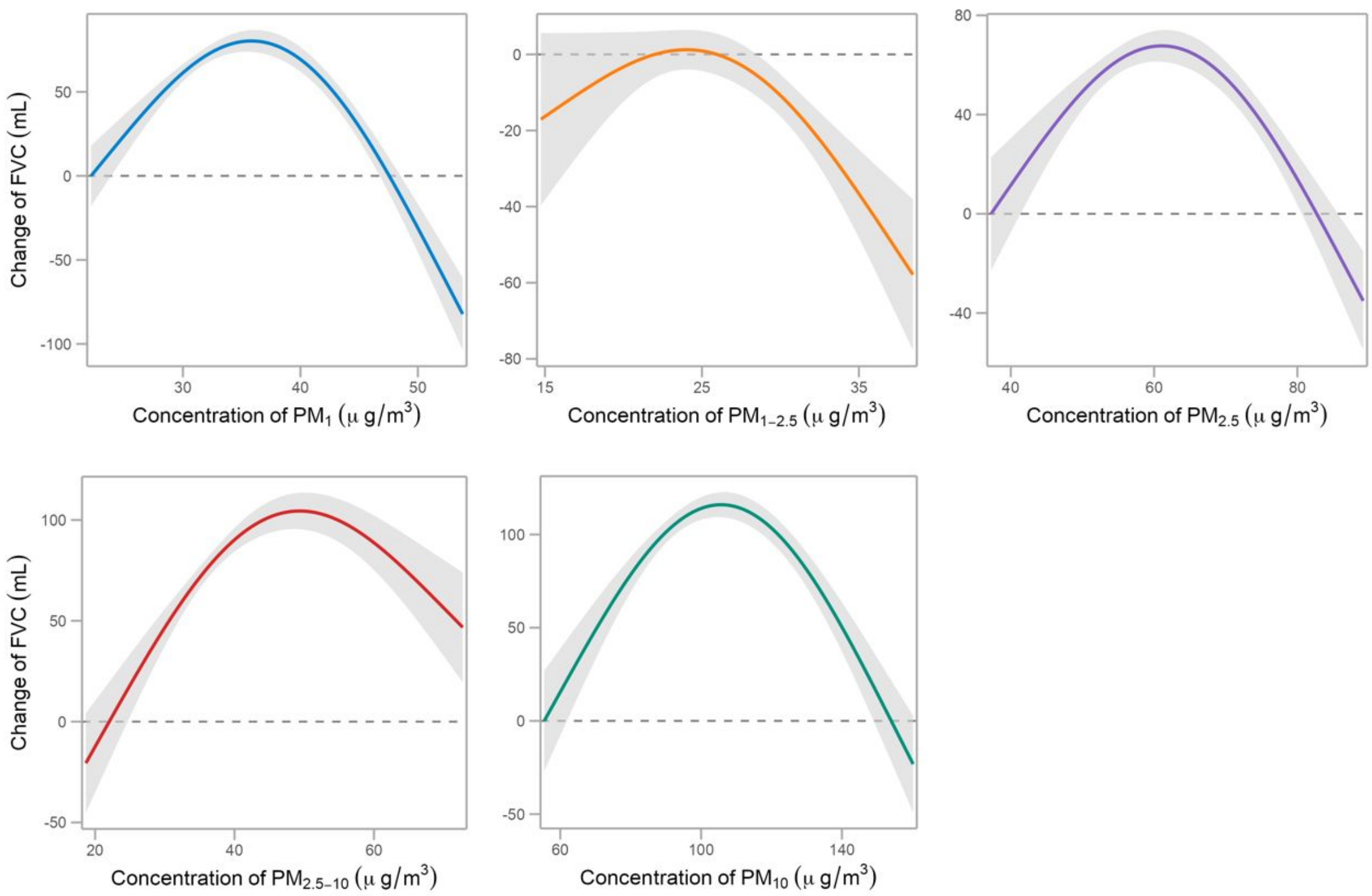

Figure S1. Exposure-response curves for the relationships of lag 1-year size-specific particulate matter concentrations with changes in FVC (mL). Abbreviations: FVC, forced vital capacity.

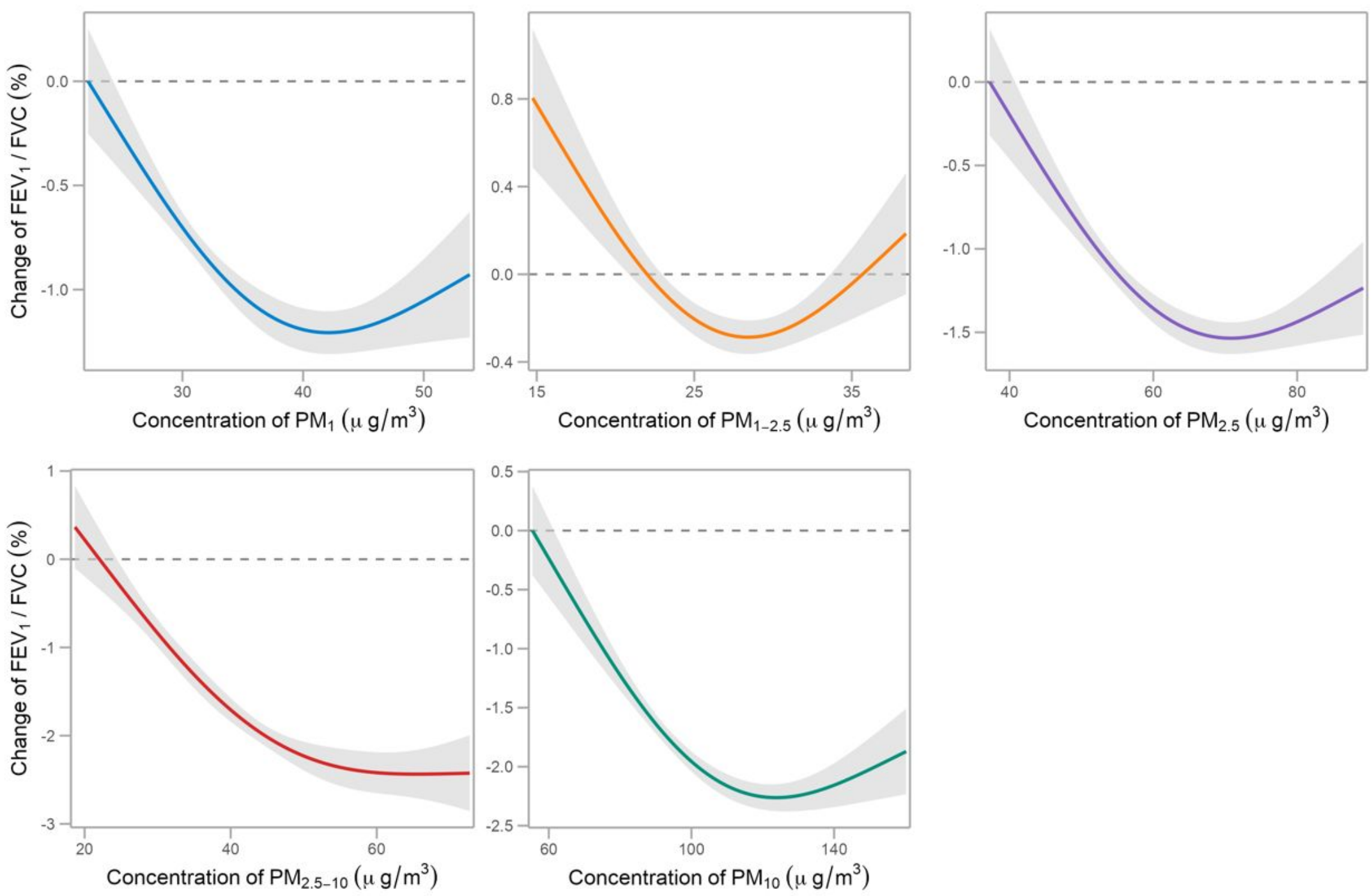

Figure S2. Exposure-response curves for the relationships of lag 1-year size-specific particulate matter concentrations with changes in FEV_1/FVC (%). Abbreviations: FEV_1, forced expiratory volume in 1 second; FVC, forced vital capacity.

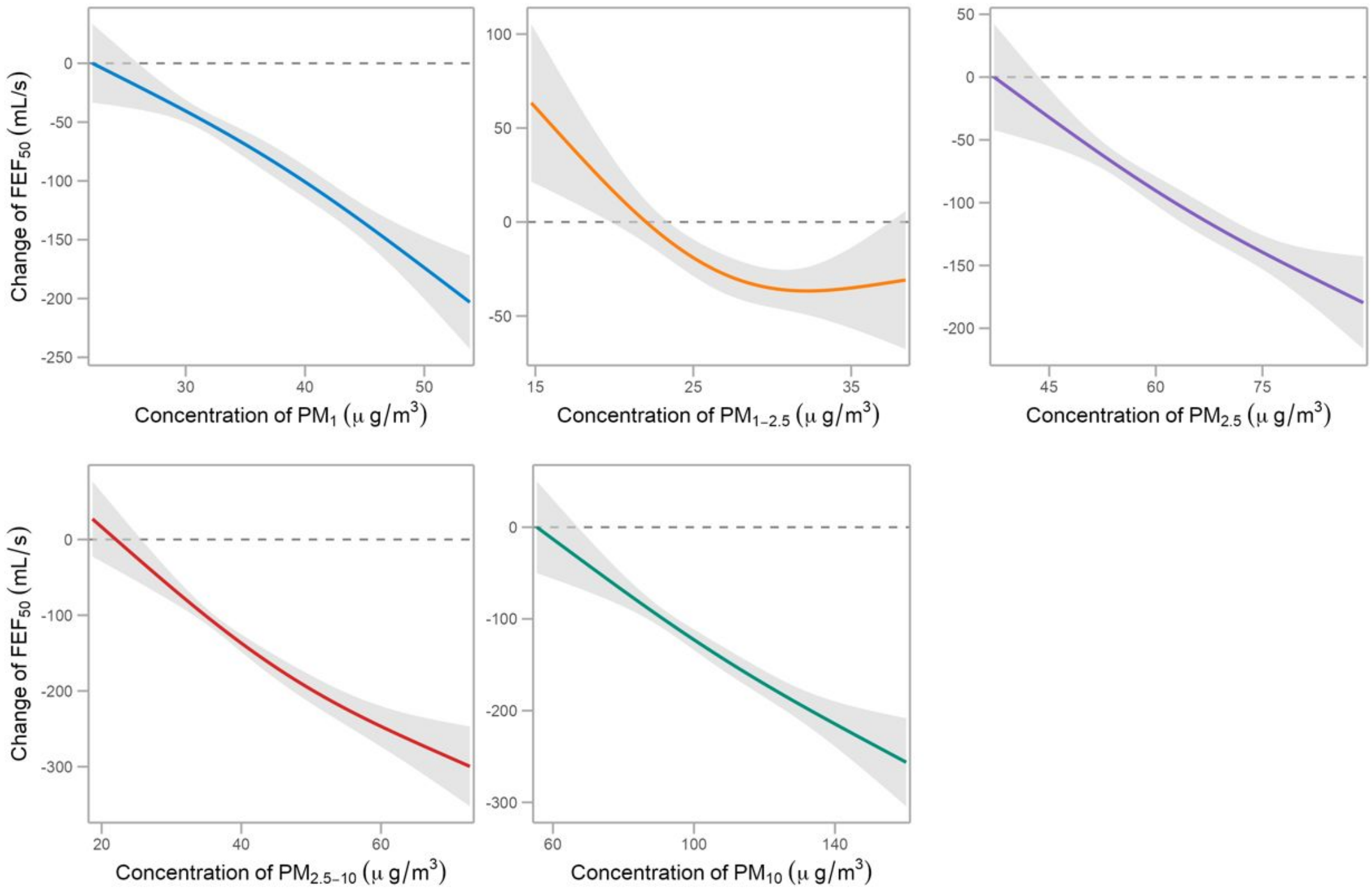

Figure S3. Exposure-response curves for the relationships of lag 1-year size-specific particulate matter concentrations with changes in FEF_{50} (mL/s). Abbreviations: FEF_{50}, forced expiratory flow at the 50th percentile of forced vital capacity.

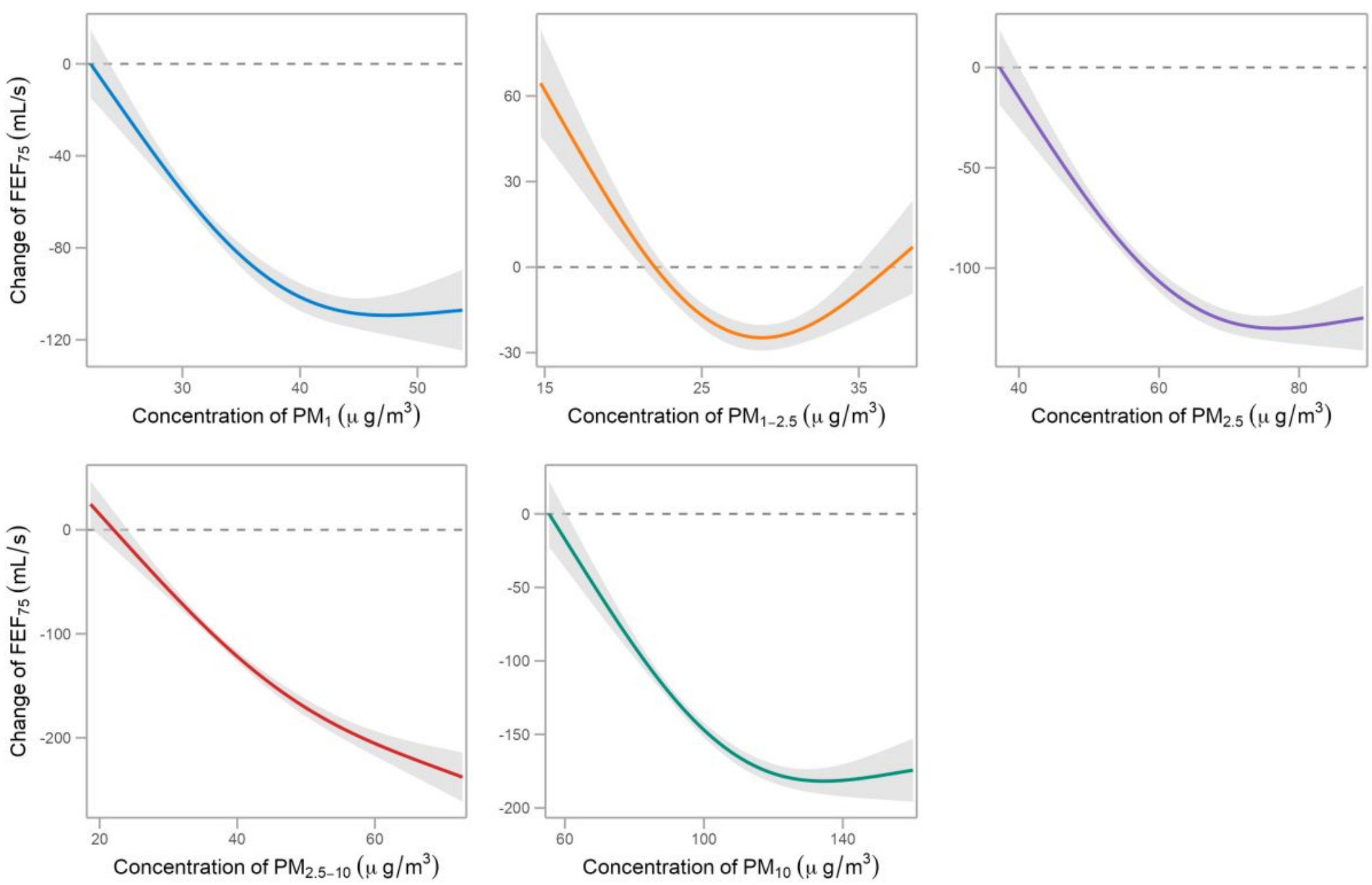

Figure S4. Exposure-response curves for the relationships of lag 1-year size-specific particulate matter concentrations with changes in FEF_{75} (mL/s). Abbreviations: FEF_{75}, forced expiratory flow at the 75th percentile of forced vital capacity.

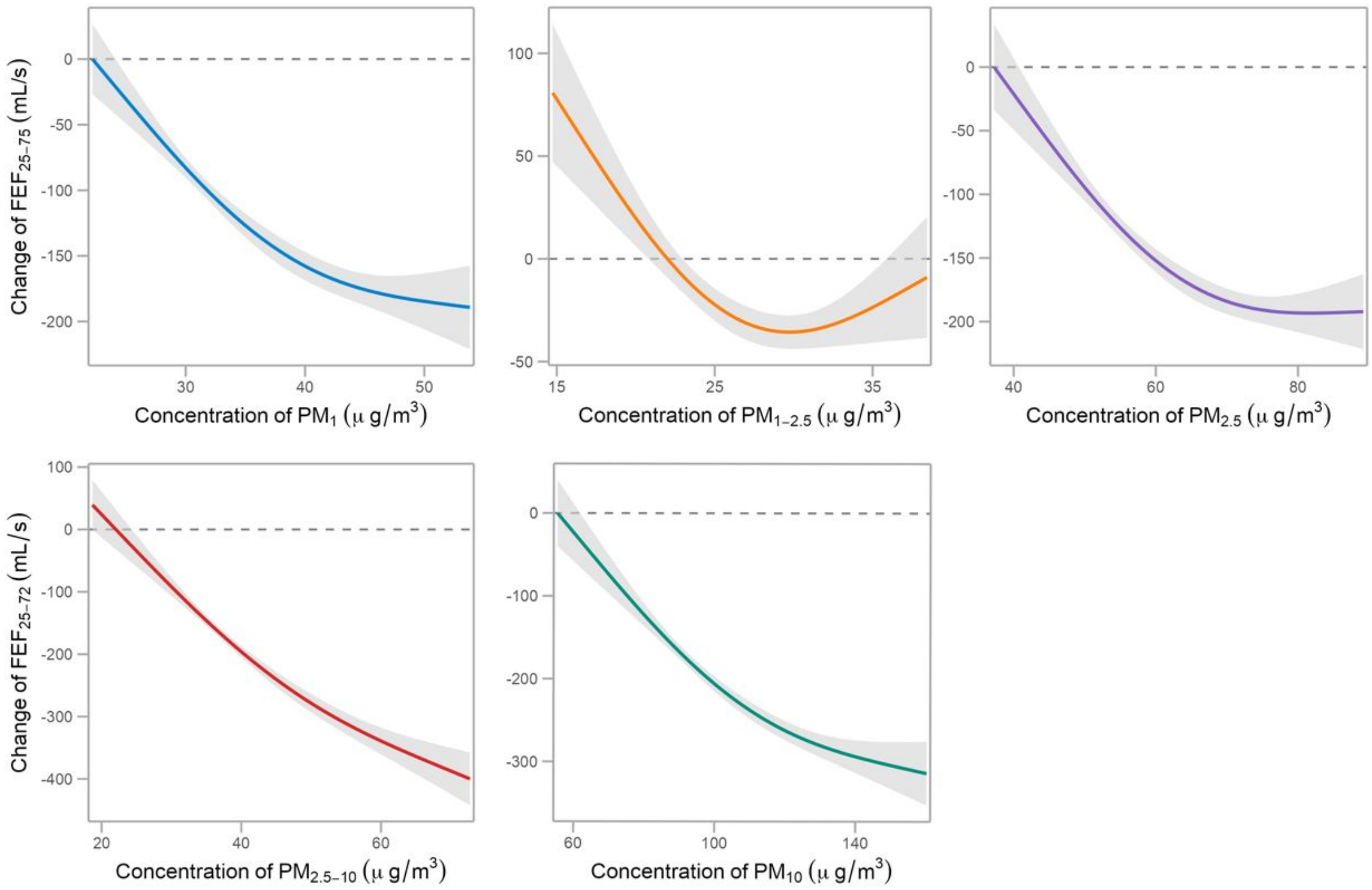

Figure S5. Exposure-response curves for the relationships of lag 1-year size-specific particulate matter concentrations with changes in FEF_{25-75} (mL/s). Abbreviations: FEF_{25-75}, forced expiratory flow between 25% and 75% of forced vital capacity.

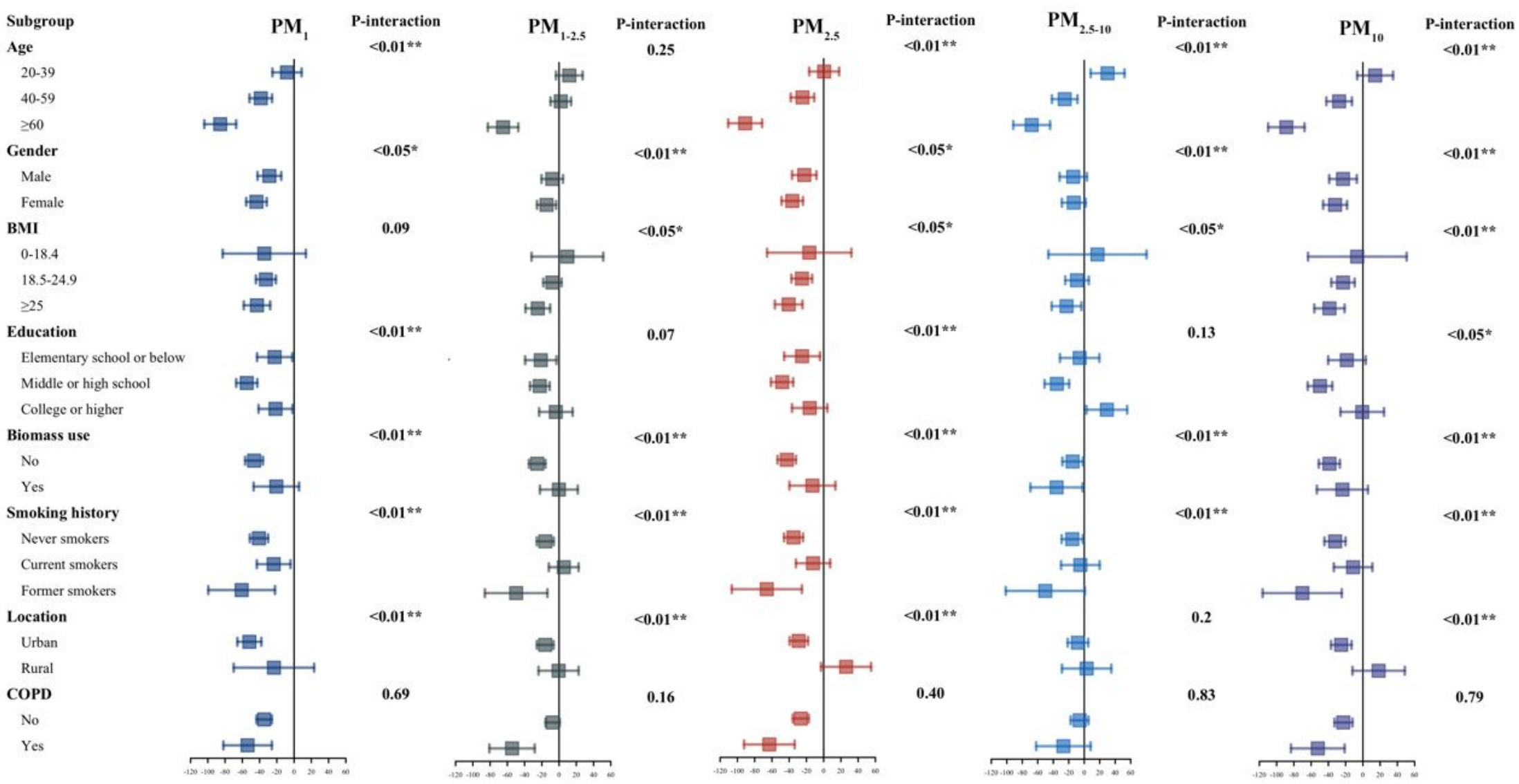

Figure S6. Changes of FEV_1 per interquartile increase of concentrations of PM_1, $PM_{1-2.5}$, $PM_{2.5}$, $PM_{2.5-10}$, and PM_{10} classified by different subgroups. Abbreviations: FEV_1, forced expiratory volume in 1 second; BMI, body mass index; COPD, chronic obstructive pulmonary disease.

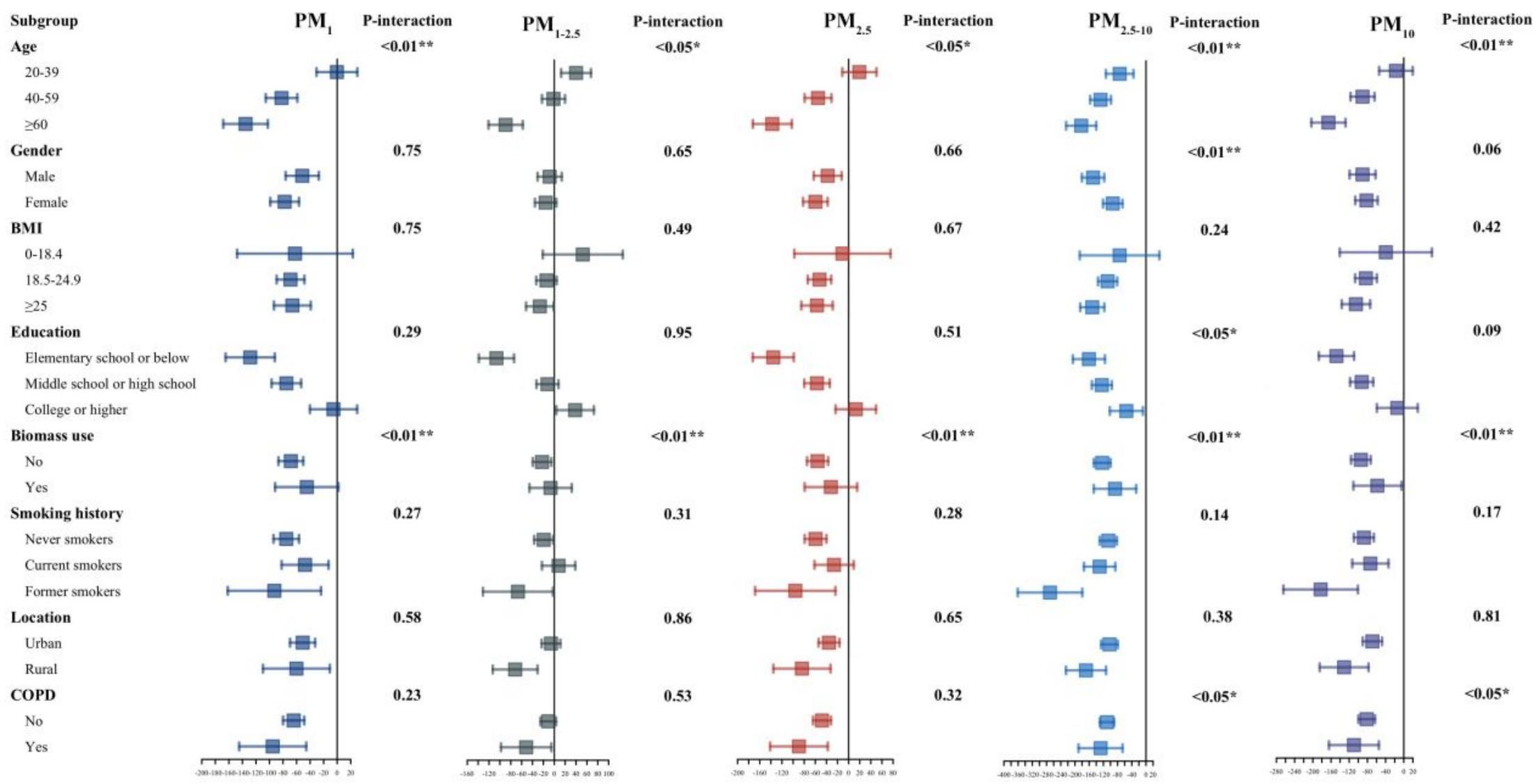

Figure S7. Changes of $FEF_{25\text{-}75}$ per interquartile increase of concentrations of PM_1, $PM_{1\text{-}2.5}$, $PM_{2.5}$, $PM_{2.5\text{-}10}$, and PM_{10} were classified by different subgroups. Abbreviations: $FEF_{25\text{-}75}$, forced expiratory flow between 25% and 75% of forced vital capacity; BMI, body mass index; COPD, chronic obstructive pulmonary disease.

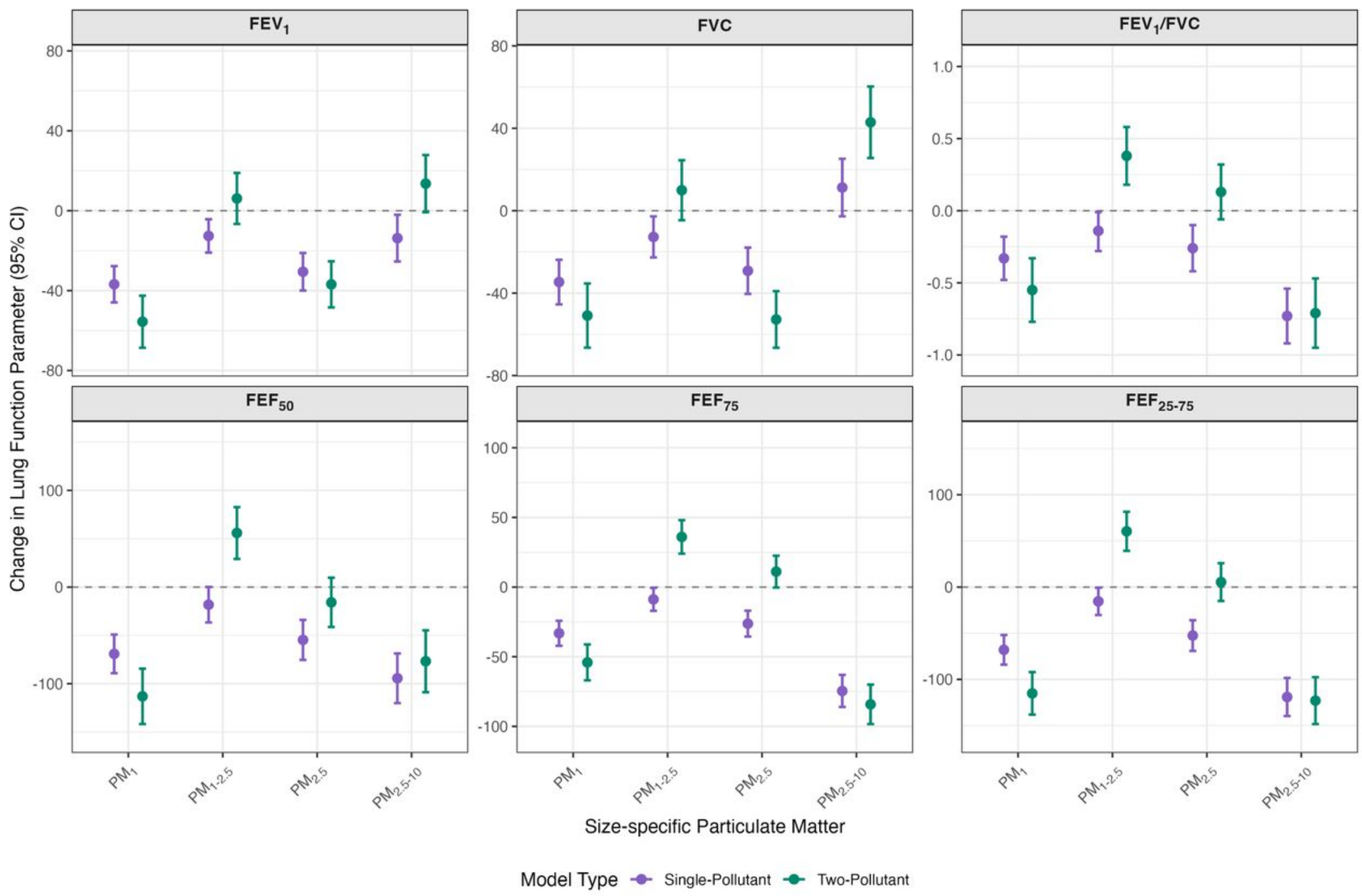

Figure S8. Associations between an IQR increase in size-specific PM and lung function measures in two-pollutant models adjusted for other PM fractions.

Data are presented as mean differences (95% confidence intervals) in mL for FEV_1 and FVC, mL/s for FEF_{50}, FEF_{75}, and $FEF_{25\text{-}75}$, and percentage points for FEV_1/FVC. All models were adjusted for age, gender, education, marriage, occupation, BMI, smoking history, smoking exposure, number of smokers in the household, biomass use, season of lung function test, geographic

region, residence, history of pneumonia or bronchitis during childhood, chronic cough during childhood, and parental history of respiratory diseases.

Each size-fraction model was mutually adjusted for a second particulate fraction: PM_1 models were adjusted for $PM_{1\text{-}2.5}$; $PM_{1\text{-}2.5}$ models were adjusted for PM_1; $PM_{2.5}$ models were adjusted for $PM_{2.5\text{-}10}$; and $PM_{2.5\text{-}10}$ models were adjusted for $PM_{2.5}$.
